# Weak Points of Double-Plate Stabilization Used in the Treatment of Distal Humerus Fracture through Finite Element Analysis

**DOI:** 10.3390/jcm13041034

**Published:** 2024-02-11

**Authors:** Artur Kruszewski, Szczepan Piszczatowski, Piotr Piekarczyk, Piotr Cieślik, Krzysztof Kwiatkowski

**Affiliations:** 1 Faculty of Mechanical Engineering, Institute of Biomedical Engineering, Bialystok University of Technology, 45A Wiejska Street, 15-351 Bialystok, Poland; akruszewski69@gmail.com; 2 Department of Traumatology and Orthopedics, Military Institute of Medicine—National Research Institute, 128 Szaserów Street, 04-141 Warsaw, Poland; piotr@msnet.pl (P.P.); pcieslik@wim.mil.pl (P.C.); inst_ort@wim.mil.pl (K.K.)

**Keywords:** distal humerus, fracture healing, stabilization, osteosynthesis, biomechanics, interfragmentary movement

## Abstract

Background: Multi-comminuted, intra-articular fractures of the distal humerus still pose a challenge to modern orthopedics due to unsatisfactory treatment results and a high percentage (over 50%) of postoperative complications. When surgical treatment is chosen, such fractures are fixed using two plates with locking screws, which can be used in three spatial configurations: either parallel or one of two perpendicular variants (posterolateral and posteromedial). The evaluation of the fracture healing conditions for these plate configurations is unambiguous. The contradictions between the conclusions of biomechanical studies and clinical observations were the motivation to undertake a more in-depth biomechanical analysis aiming to indicate the weak points of two-plate fracture stabilization. Methods: Research was conducted using the finite element method based on an experimentally validated model. Three variants of distal humerus fracture (Y, λ, and H) were fixed using three different plate configurations (parallel, posterolateral, and posteromedial), and they were analyzed under six loading conditions, covering the whole range of flexion in the elbow joint (0–145°). A joint reaction force equal to 150 N was assumed, which corresponds with holding a weight of 1 kg in the hand. The biomechanical conditions of bone union were assessed based on the interfragmentary movement (IFM) and using criteria formulated by Steiner et al. Results: The IFMs were established for particular regions of all of the analyzed types of fracture, with distinction to the normal and tangential components. In general, the tangential component of IFM was greater than normal. A strong influence of the elbow joint’s angular position on the IFM was observed, with excessive values occurring for flexion angles greater than 90°. In most cases, the smallest IFM values were obtained for the parallel plaiting, while the greatest values were obtained for the posteromedial plating. Based on IFM values, fracture healing conditions in particular cases (fracture type, plate configuration, loading condition, and fracture gap localization) were classified into one of four groups: optimal bone union (OPT), probable union (PU), probable non-union (PNU), and non-union (NU). Conclusions: No plating configuration is able to ensure distal humerus fracture union when the full elbow flexion is allowed while holding a weight of 1 kg in the hand. However, flexion in the range of 0–90° with such loadings is acceptable when using parallel plating, which is a positive finding in the context of the early rehabilitation process. In general, parallel plating ensures better conditions for fracture healing than perpendicular plate configurations, especially the posteromedial version.

## 1. Introduction

Distal humeral fractures (DHF) represent about 30% of the fractures involving the humerus [1,2], and they are the cause of about 37% of all elbow surgeries [3]. The gold standard in DHF surgical treatment is open reduction and internal fixation (ORIF), which uses two locking plates and a set of screws [4,5]. However, the use of double-plating in DHF osteosynthesis still results in a high complication rate, estimated to affect over 50% of all operated patients [6,7,8,9]. The most common complications are the need for reoperation due to, for example, deep infection or painful implant (20.8–49%); non-union, occurring when the fracture is not clinically or radiographically united after 6 months of fixation (4.1–9.3%); stiffness of the elbow joint, diagnosed, for example, when the patient cannot achieve a 30°–130° arc (19–46.5%); or degenerative changes, e.g., osteoarthritis (9–21.1%) or heterotrophic ossifications (5.1–21.8%). In this situation, elbow arthroplasty is increasingly used as an alternative, though much more radical, DHF treatment method [10]. Postoperative complications after the use of double-plating may have various causes and result from the course of the procedure itself, the specificity of the surgical approach, or coexisting diseases. However, non-union, limitations in joint movement, and heterotrophic ossifications are probably related to the insufficient stability of the bone fragments and improper joint movement during early postoperative rehabilitation. It is known, however, that stabilization should provide stable-enough fixation to obtain a union. It also should allow for an early rehabilitation process, as movement is essential for success in the final treatment due to the fact that the elbow is intolerant to immobilization [11,12,13].

Nowadays, there are two popular plating techniques used to treat distal humerus fractures. The first one involves parallel plating with medial and lateral plates [14,15], while the other involves perpendicular plating [16] and has two available options: “posterolateral”, with medial and posterolateral plates, and “posteromedial”, with lateral and posteromedial plates. Parallel plating is the consequence of earlier reports of unsatisfactory results among patients with perpendicular plating (the standard proposed by AO/ASIF) [17]. However, the optimal plate configuration still remains controversial.

Biomechanical studies attempted to assess the stability of the fixation of distal humerus fractures, and they were based primarily on the evaluation of the global stiffness of the bone–plate system. Most of the discussed studies indicated the advantage of parallel plating [18,19,20]. Both perpendicular configurations usually ensure the necessary stiffness of the fixation as well, but in general, their mechanical parameters are worse than those of parallel plating [21,22]. As a result, some contradictions can be noticed between the conclusions formulated in biomechanical studies and clinical observations. However, better clinical results are reported in the case of perpendicular plating [23,24,25,26], which is inconsistent with the fact that the parallel plating is indicated to guarantee more rigid stabilization. It seems that the biomechanical studies did not encompass all clinically important aspects of the problem. Ambiguities in the assessment of plate configurations may largely result from limitations of the testing method, such as oversimplified loading conditions (for example, only axial or bending loadings) [27,28]. The other problem is the lack of realistic analysis of interfragmentary movement in multi-comminuted fractures and the assessment of fixation only on the basis of global stiffness [21,27]. It is well known, however, that for proper bone union, it is crucial to stabilize all bone fragments to avoid their mutual movement, and the assessment of global stiffness does not provide a realistic evaluation of the union conditions when it comes to the particular bone fragments. All of these limitations may be the reason for the abovementioned contradictions between the biomechanical and clinical assessment of particular DHF stabilization methods. This was the motivation to undertake the present research.

The aim of the present study is to present a more comprehensive evaluation of biomechanical conditions of distal humerus fracture healing and, based on the results obtained, provide an indication of the weak points of particular variants of double-plating for such fractures. This analysis should allow for more optimal DHF treatment by raising awareness of the choice in plate configuration and introducing necessary restrictions during fracture healing and rehabilitation.

## 2. Materials and Methods

In order to achieve the above-presented research goal and taking into account the limitations of previous analyses, three main assumptions were made when planning the experiment. (1) The interfragmentary movement (IFM) of particular pairs of bone fragments should be used to calculate the local stiffness of the bone union and thus assess the biomechanical conditions of fracture healing based on Steiner’s analysis [29]. (2) Research should be conducted based on realistic geometrical structures of typical DHFs present in clinical practice. (3) Finally, loading conditions occurring throughout the entire range of elbow flexion–extension should be taken into consideration.

Both parallel and perpendicular plate configurations, distinguishing the latter’s posteromedial (PM) and posterolateral (PL) versions, were used as the objects of the research.

**Figure 1:**
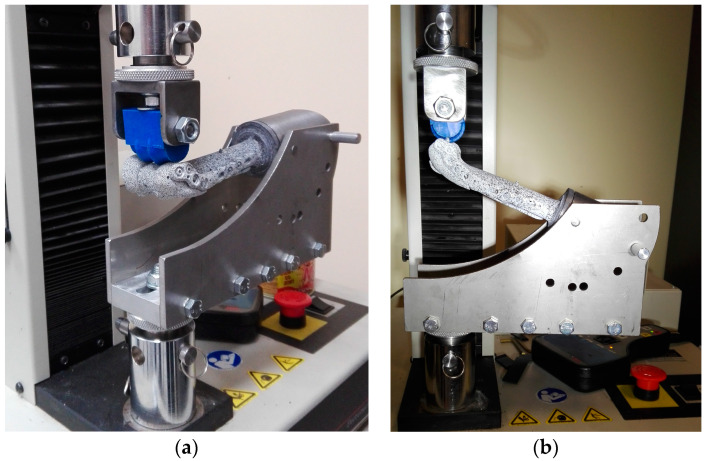
Measuring station consisting of the MTS Insight 1 kN testing machine, a special clamping device, and the ARAMIS digital image correlation system: (**a**) general view; (**b**) side view of the device enabling the loading of the sample at various angles.

Modeling and numerical simulation were performed using the finite element method (FEM) as the main research method. However, a laboratory experiment was undertaken using an artificial humeral bone and testing machine to validate the numerical models and to obtain some parameters for numerical simulations (Figure 1).

**Figure 2:**
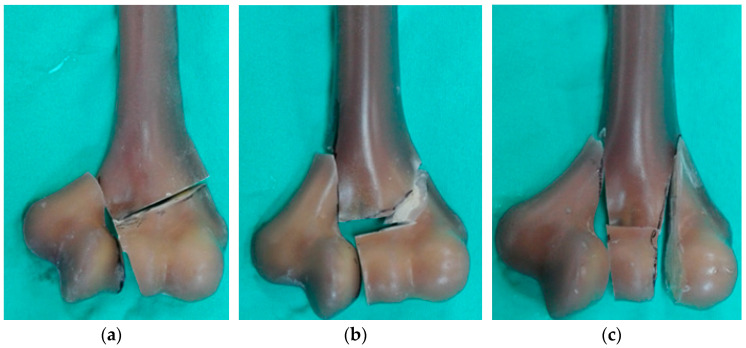
Three types of DHFs included in the study, determined based on Jupiter and Mehne’s classification: (**a**) Y fracture, (**b**) λ fracture, and (**c**) H fracture.

The same geometry of the humerus was used, both in the experimental and the numerical studies, using composite humeral bone (Sawbones Europe AB, Malmo, Sweden, 4th Gen., Composite, 17 PCF Solid Foam Core, Large). The three geometric variants of the fracture most frequently occurring in clinical practice were included (Figure 2) and marked Y, λ, and H according to the DHF classification proposed by Jupiter and Mehne [30]. The modeled gap between the particular bone fragments was about 1.6 mm wide.

**Figure 3:**
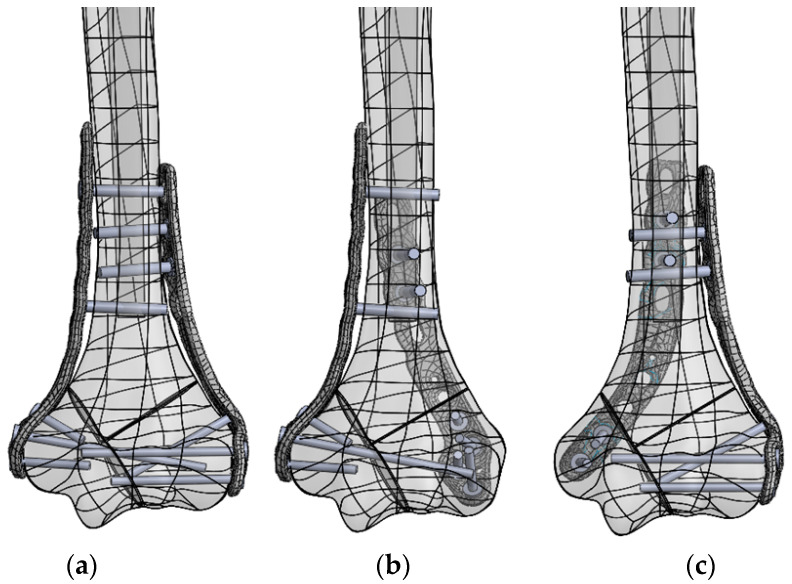
Placement of plates and screws in the Y-type fracture for the following plate configurations: (**a**) parallel, (**b**) posterolateral, and (**c**) posteromedial.

Particular models of the fractured bone were fixed using the VariAx Elbow Plate System (Stryker, Portage, MI, USA) made of titanium alloy, reproducing the three abovementioned spatial plating configurations: parallel, posteromedial, and posterolateral. The number and localization of the screws connecting the plates to the bone were modeled based on their implantation in clinical practice. General rules for inserting screws according to AO guidelines in perpendicular plating and principles for the optimization of stability postulated by O’Driscoll, applicable mainly for parallel plating, were used for the screw placement [14,27]. An example of the screw arrangements is presented in Figure 3.

For the numerical analysis, bone models and particular plates were scanned using an optical scanner (Atos Core 200, GOM, Braunschweig, Germany), and their finite element models were obtained using CAD/CAE software (ANSYS Workbench 2021 R1, Canonsburg, PA, USA). The screws connecting the plates to the bone model were simplified in the numerical analysis and modeled without threads. The screws in the area of the humeral shaft were modeled as a cylinder with a diameter of 2.75 mm, which corresponds to the core diameter of the screw used in experimental setup with an outer thread diameter of 3.5 mm; those intended for the distal end of the humerus were modeled as cylinders with a diameter of 2 mm, which corresponds to the core diameter of the screw with an outer thread diameter of 2.7 mm.

**Figure 4:**
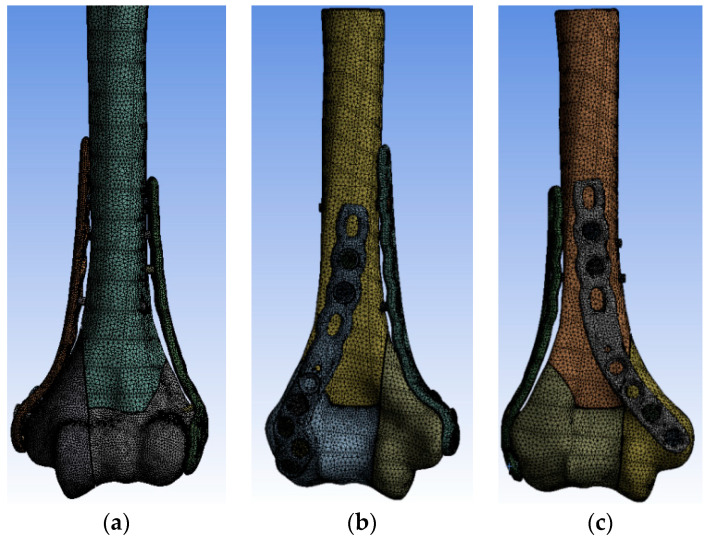
FEM model for λ fracture fixed with (**a**) parallel, (**b**) posterolateral, and (**c**) posteromedial plate configurations.

Discretization was performed using the 10-node tetrahedral element Solid187. Convergence of the solution was ensured by diminishing the size of the elements up until the change in the maximum equivalent stress did not exceed 5%. The final models consisted of 380–420 thousands of elements and 230–280 thousands of nodes (Figure 4).

### 2.1. Loads and Boundary Conditions

During the laboratory experiment (Figure 1), the bone was mounted using special equipment in six different angular positions in relation to the load axis of the testing machine. This way, it was possible to reconstruct variable directions for the joint reaction force (JRF) vector in the humeroulnar joint during the elbow flexion movement in its entire range (0–145°). The loading directions for particular joint angles were assumed based on Kincaid and An’s analysis [31] (Table 1). In all cases, the same value of JRF, equal to 150 N, was used, which corresponds to the loads occurring when holding a weight of approximately 1 kg in the hand with the elbow flexed at a 90° angle.

The value of the displacements of the testing machine’s compressing upper plate, recorded during the laboratory experiment for particular loading directions, was used as kinematic boundary condition used in the numerical simulations. In order to validate the numerical model, the displacements of selected points located on the plates were recorded during the laboratory experiment using the digital image correlation (DIC) technique, and then they were compared with their numerically determined values. The obtained differences did not exceed 3.5%.

### 2.2. Material Properties

The material model took into account the heterogeneous structure of the humerus, which was divided into cortical and spongy tissue. The bone shaft was built of cortical tissue with a reconstructed medullary cavity. In the epiphyseal and metaphyseal regions, the external part was modeled as cortical bone, while the internal part was modeled as spongy bone. The thickness of the outer layer corresponding to the cortical tissue in this region was about 2 mm. The values of the material parameters used in the model were as follows (elastic modulus; Poisson coefficient): cortical bone (16.7 Gpa; 0.34), spongy bone (0.155 Gpa; 0.34) [32], and titanium alloy (110 Gpa; 0.30).

### 2.3. Interfragmentary Movement

**Figure 5:**
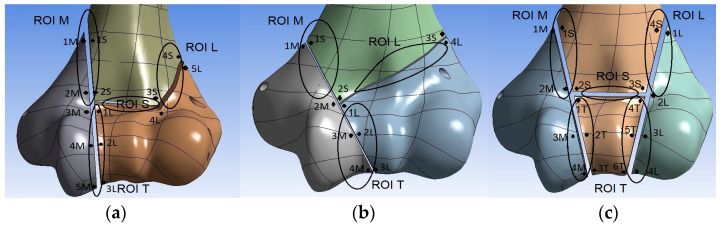
Pairs of points and their distribution in individual bone fragments in fractures: (**a**) λ; (**b**) Y; and (**c**) H. Front view; analogous pairs of points are marked on the back side (not visible).

A set of points was evenly distributed around the circumference of each pair of bone fragments in the models, where the points located on one side of the fracture gap had their counterparts on the other side (Figure 5). Then, the displacements of each point in the local coordinate system were determined and the mutual displacements between the pairs of points were calculated, distinguishing between displacements in the normal and tangential directions. The values of the mutual displacements for all pairs of points around particular fracture gaps are provided in Table S1 in Supplementary Material. Assuming that the risk of non-union is determined by the least favorable conditions occurring in the entire fracture gap, the greatest value of the mutual displacements between all pairs of points located in particular region of interest (ROI) were taken for further analyses, named the interfragmentary movement (IFM). Four ROIs were defined: ROI M: the fracture gap between the shaft and the medial bone fragment; ROI L: the fracture gap between the shaft and the lateral bone fragment; ROI S: the fracture gap between the shaft and the trochlea (only in λ- and H-type fractures); and ROI T: the fracture gaps inside the trochlea region. In H-type fractures, the IFM for ROI T was taken as the largest displacement value in the whole trochlea region (Figure 5c).

### 2.4. Assessment of the Biomechanical Conditions of Fracture Healing

With the research aim of identifying weak points of fracture fixation using particular plate configurations, we assumed that the assessment of the biomechanical conditions of fracture healing should indicate cases with high risk of bone non-union. On the other hand, it is known that bone union should occur in cases where the level of interfragmentary movement remains within a certain range of values, ensured by the appropriate stiffness of the stabilization. Based on Steiner’s analyses, it was assumed that the optimal axial stiffness of the stabilization promoting bone union should be in the range between 1000 N/mm (lower limit) and 2500 N/mm (upper limit) [29]. In the case of the elbow joint’s reaction force equaling 150 N during the test, the optimal value of the axial component of IFM should be in the range of 0.06–0.15 mm. In turn, the lower limit of the bone-plating stiffness in the tangential direction should reach 400 N/mm for a gap of 1 mm. When the load value is equal to 150 N, the upper limit of the acceptable tangential component of IFM is 0.375 mm. We assumed that the optimal conditions for bone union (OPT) occur when the axial component of IFM remains in the range of 0.06–0.15 mm while the tangential component is below 0.375 mm. The previously mentioned research also shows that IFM values lower and higher than the normal optimal value could delay bone union, but they do not always lead to bone non-union. The coexisting range of the tangential IFM is, however, crucial. For this reason, we assumed that the normal component of IFM outside of the optimal range (below 0.06 mm or in the range of 0.15–0.375 mm) together with its tangential component below 0.375 mm would ensure a potentially non-optimal biomechanical condition, but bone union is still probable (classified as “probable union”—PU). An increase in IFM of over 0.375 mm could be treated as an increased risk of non-union, and those results are evaluated as a “probable non-union”—PNU. According to Steiner’s analyses, the limit of tangential stiffness for a wider fracture gap (3 mm) decreases to 300 N/mm, which results in a greater limit of IFM equal to 0.5 mm. In this context, we assumed that fracture stabilization ensuring an IFM value below 0.5 mm (in any direction) cannot be treated as a cause of bone non-union when the loading is equal to 150 N. This way, contrary to the above assumption, IFM values higher than 0.5 mm were assumed to be a biomechanical condition with reasonable risk of bone non-union (NU).

**Figure 6:**
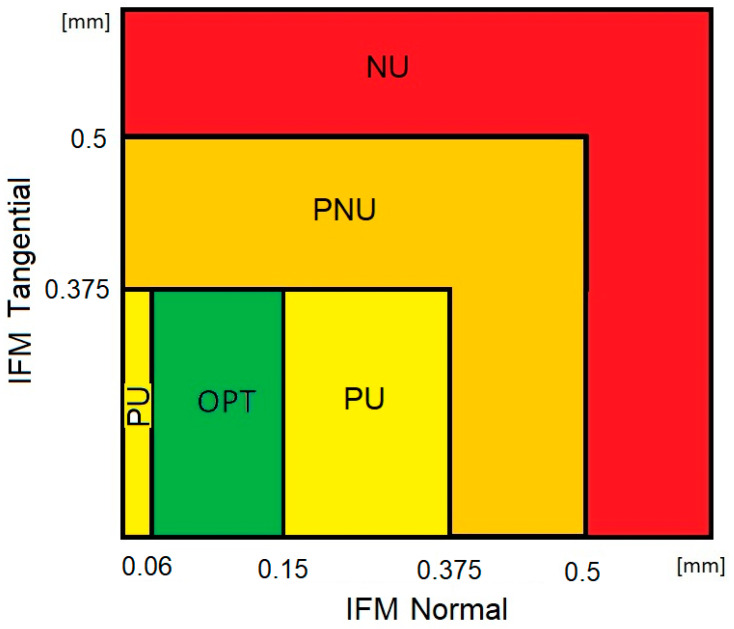
Criteria for assessing the biomechanical conditions of fracture healing for a 150 N load acting on the bone-plating system: OPT—optimal bone union conditions; PU—high probability of achieving bone union; PNU—probable non-union; NU—very high risk of non-union.

Finally, in order to assess the biomechanical condition of fracture union, particular cases (combinations of plate configurations, loading directions, fracture type, and ROI) were classified into one of four groups based on the obtained IFM values and the assumptions presented above (Figure 6):

– Optimal bone union (OPT)—the value of the normal component of IFM within the range of 0.06–0.15 mm and the value of the tangential component of IFM below 0.375 mm;
– Probable union (PU)—the value of normal displacements in the range of 0–0.06 mm or 0.15–0.375 mm and the value of tangential displacements below 0.375 mm;
– Probable non-union (PNU)—the value of both tangential and normal displacements greater than 0.375 mm but below 0.5 mm;
– Non-union (NU)—the value of normal or tangential displacements greater than 0.5 mm.

Summing up the presented methodology, it is worth noting that the basis of the research was the numerical analysis with use of the finite element method, carried out using models validated on the basis of experimental results. It should be emphasized that nine combinations of the bone–plate system (three variants of the DHF, fixed with one of three plate configurations) were analyzed under loads acting in six directions corresponding to the full range of elbow flexion. The analysis resulted in fifty-four spatial variants of the model. The biomechanical conditions of fracture healing were evaluated based on the values of interfragmentary movement determined in four regions of interest, covering the entire fracture region. The fracture union conditions were classified based on Steiner’s analyses as optimal (OPT), highly probable union (PU), probable non-union (PNU), and risk of non-union (NU).

## 3. Results

Taking into account the analyzed variants of the model discussed above (including fracture types, plate configurations, and loading directions) as well as the four regions of interest (Figure 5) and two components of interfragmentary movement (normal and tangential), a substantial dataset was obtained for analysis. For this reason and for a concise presentation, the results are shown mainly in graph form. This should allow for comparative analyses of the influence of particular factors on IFM values. Additionally, the most important findings are briefly described after graphical presentation. For clarity, the same range of IFM values is maintained on all graphs. In the second part of the presentation of the results, particular variants of the model are classified in terms of their assessed biomechanical bone union conditions based on the IFM values obtained using the methodology discussed earlier (Figure 6).

**Figure 7:**
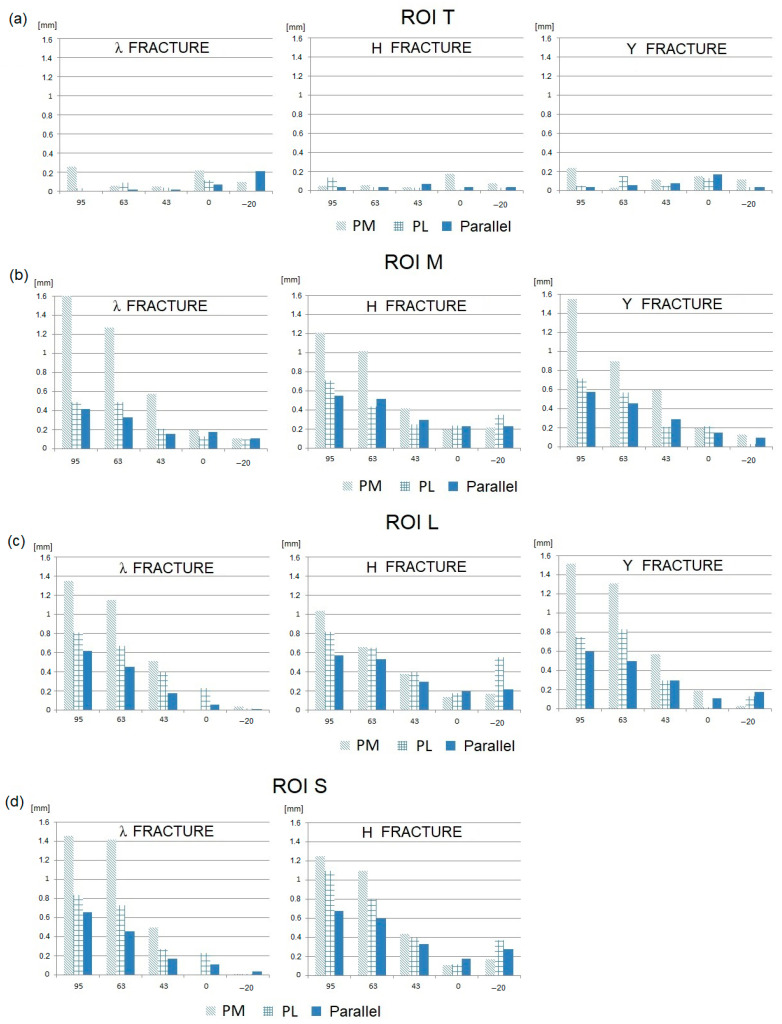
IFMs in the tangential direction for three plate configurations with respect to the JRF direction: (**a**) ROI T; (**b**) ROI M; (**c**) ROI L; and (**d**) ROI S.

**Figure 8:**
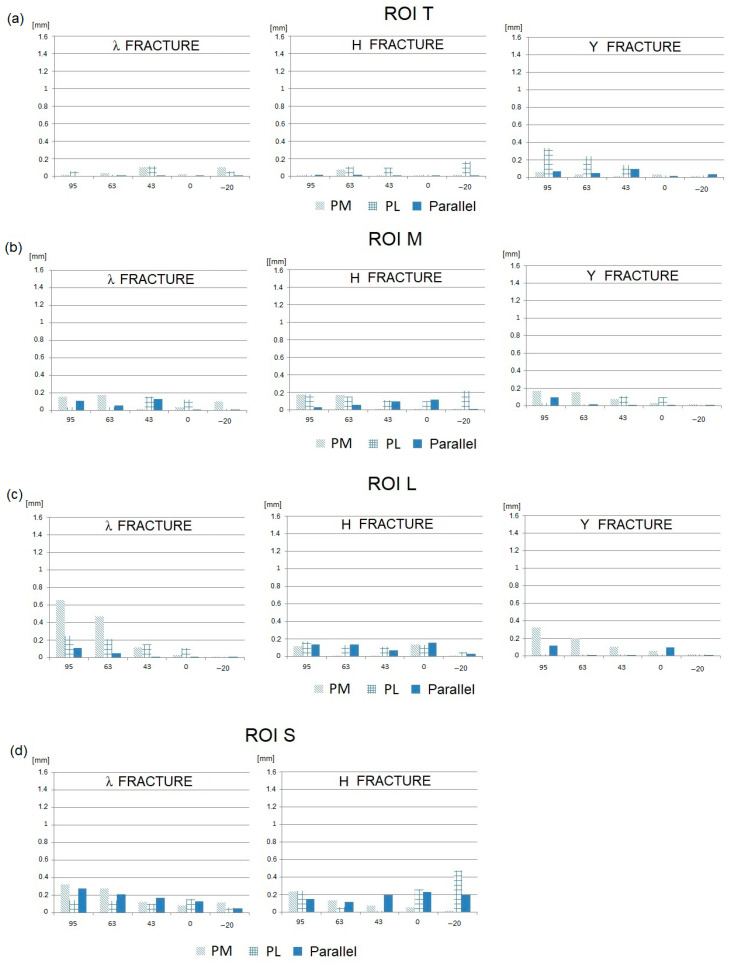
IFMs in the normal direction for the three plate configurations with respect to the JRF direction: (**a**) ROI T; (**b**) ROI M; (**c**) ROI L; and (**d**) ROI S.

Then, the IFM values obtained for the three analyzed fractures, all spatial plate configurations, and the six loading conditions (JRF directions) are presented in Figure 7 (tangential component) and Figure 8 (normal component).

Analyzing the presented results of the numerical research, the following phenomena can be observed:

– In general, the tangential components of IFMs are significantly greater than the normal components.
– The smallest IFMs, both tangential and normal, are observed for the parallel plate configuration in the majority of fracture types and elbow joint flexion angles.
– In most cases, the largest IFMs are observed for posteromedial (PM) stabilization.
– The angular position of the elbow joint and the related direction of the joint force reaction has a very strong influence on the value of IFM. It can be observed that the maximum IFM values occur when the elbow joint is almost fully flexed (JRF direction 63–95°; joint angle 120–145°) in all plating configurations and all types of fractures.
– For elbow joint angles in the range of 0–90° (JRF direction −20–43°), the IFM values are relatively low. In this angular range, the differences in the IFM values obtained for different plating configurations and different types of fractures are somewhat unclear.

The mutual displacements of bone fragments also depend on the type of fracture, although this effect is not as pronounced as in the case of the elbow flexion angle (or JRF direction) and the spatial configuration of the plates. The influence of the type of fracture on the IFM value results primarily from its position in the space of individual fracture gaps in relation to the line of screw insertion and the localization of the particular plates.

Based on both the normal and tangential IFM component values in a particular ROI, biomechanical conditions of fracture healing were evaluated for all cases (analyzing combinations of plate configurations, elbow angles, and fracture types). These are presented in Table 2.

Analyzing the obtained results, we can conclude that the following cases are classified as having a high risk of bone non-union:

– All variants of the fracture gap except in the trochlear region (i.e., the S, M, and L regions) in all types of fractures (λ, Y, and H) stabilized in a perpendicular configuration, both posterolateral (PL) and posteromedial (PM), for an elbow flexion angle equal to or greater than 120°;
– All variants of the fracture gap except in the trochlear region (i.e., the S, M, and L regions) in all types of fractures (λ, Y, and H) stabilized in a parallel configuration, for the maximum elbow joint flexion angle (145°),
– The fracture gap between the lateral fragment and the shaft (region L) in Y- and H-type fractures when they are stabilized in a parallel configuration, and when the elbow joint is flexed to 120°;
– The fracture gap between the medial fragment and the shaft (region M) in Y-type fractures with stabilization in a posteromedial (PM) configuration, for an elbow flexion angle of 60–90°.

The positive exception to the above rules is the fracture gap between the medial fragment and the shaft (region M):

– In the case of a λ-type fracture stabilized in a posterolateral (PL) or parallel configuration, the chance of union is not eliminated in the entire angular range of the loading direction;
– In the case of an H-type fracture stabilized in a posterolateral (PL) configuration, a high probability of non-union is obtained only for the maximum elbow flexion (145°).

A reasonable probability of non-union (PNU) also occurs in the following instances:

– In the case of stabilization in a posteromedial (PM) configuration for all types of fractures and all fracture gaps, for elbow joint flexed at 60° or 90°, with the exception of the gap between the lateral fragment and the shaft (ROI L) in a Y-type fracture;
– In the case of stabilization in a parallel configuration, for the elbow joint flexed at 120° in following situations:
  – In the gap between the medial fragment and the shaft (ROI M) for Y- and H-type fractures;
  – In the gap between the lateral fragment and the shaft (ROI L) for λ-type fractures;
  – In the gap between the trochlea and the shaft (ROI S) for H-type fractures.

In conclusion, based on the presented results, none of the stabilization variants provide the conditions necessary to achieve the union of intra-articular, multi-comminuted distal humerus fractures if a full range of motion is allowed in the elbow joint. Excessive mobility at the distal end of the humerus relative to the shaft of the bone is visible when the elbow joint is fully flexed in virtually every type of fracture and plate configuration. When the plating is used in a perpendicular configuration, this effect also occurs when the elbow is flexed at 120°, and in the posteromedial configuration, in some cases, it occurs even at 60° of flexion.

## 4. Discussion

The aim of this study was to indicate the weak points of the stabilization used during the surgical treatment of distal humerus fractures. These results should allow for the better understanding of the frequently occurring serious complications observed in clinical practice during fracture healing. It is possible to state that this aim was achieved. In the summary of the results presented above, we justify the clinical problems in DHF stabilization. The source of the problem may be excessive interfragmentary movement occurring when the full range of elbow joint flexion is allowed, together with the joint loading reaching 150 N. We show that, in this situation, none of the stabilization variants provide the sufficient fixation stability necessary to achieve the union of intra-articular, multi-comminuted distal humerus fractures. As mentioned earlier, ensuring an appropriate level of bone fragment mutual displacement is one of the key conditions for achieving proper bone union [33,34]. In this situation, the excessive mutual displacement of the fragments may result in bone non-union, causing frequent complications in DHF treatment [7,8,35]. Helfet et al. [36] analyzed the treatment outcomes of patients with previous distal humerus fracture non-union. They noted that 75% of cases were the result of failed internal fixation. Failure to adhere to the rigid stabilization of the lateral and medial column of the distal humerus with fixator plates can dramatically increase the rate of non-union complications by up to 75% [37].

It should be emphasized that this effect in the presented results was achieved by allowing for a relatively small reaction value in the joint, corresponding to lifting approximately 1 kg with the hand. This effect is seen for all stabilization cases, regardless of the type of fracture. This effect becomes more significant when the plates are used in a perpendicular configuration, especially when the posteromedial (PM) version is chosen. In this case, the presented results indicate a high risk of non-union even if a flexion of 60 degrees is allowed. This is consistent with the observations presented by Ku et al. [12] and Shin et al. [23], who indicated a higher rate of non-union in the case of stabilization in a perpendicular configuration. Excessive mobility at the distal end of the humerus relative to the shaft of the bone can result when the plates are used in a perpendicular configuration since the plates work asymmetrically. This leads to an increase in the mutual displacement of bone fragments, especially in their tangential component. The results for the posteromedial configuration are worse than those for the posterolateral configuration due to the lower stiffness of the posterior plate used in particular variants of the perpendicular plating system. This is influenced by unfavorable posteromedial plate geometry and its position in relation to the loading direction. Penzkofer et al. [18] presented a similar effect indicating worse healing conditions when using a posteromedial plate orientation for a flexed elbow joint.

Some clinical results indicate a relatively lower overall rate of complications when using plates in a perpendicular configuration. This is most likely due to the lower invasiveness of this surgical technique. At the same time, there is reason to state that when a perpendicular configuration is used, the most serious complication, i.e., the non-union of a broken bone, is more common [7]. While the installation of a perpendicular plate configuration itself carries a lower risk of complications, e.g., related to damage to nerves or blood vessels, the plating system may not provide sufficiently stable conditions for the union of the bone fragments.

We obtained relatively better results for stabilization with a parallel plate configuration, although this variant also does not ensure proper union conditions when movement is allowed throughout the entire range of elbow flexion. These better results obtained with a parallel configuration may be due to the more favorable space orientation in the plates, which are positioned parallel to each other and preferably in relation to the direction of the acting force (larger cross-sectional dimension of the plates set parallel to the plane of the force action). This effect is consistent with the results presented by Zha et al. [28]. It is also worth noting that the parallel arrangement of the plates allows for the use of maximum-length screws connecting the plates to as many bone fragments as possible, which additionally reduces their mutual displacement. This is consistent with the clinical results reported in the literature. O’Driscol [14] analyzed the clinical outcomes of humerus fractures and concluded that the parallel plate arrangement provided better fracture stabilization than the perpendicular configuration. Jung et al. [17] pointed out that it is possible to use the triangular stabilization technique for two-column reconstruction only with the use of parallel plates. This method ensures a mechanical connection between the lateral and the medial columns through the trochlea. This is described as effective and reliable in the treatment of intra-articular fractures of the distal humerus. This technique should increase the chance to obtain adequate stabilization in both the trochlea region and the medial and lateral columns. However, as mentioned earlier, based on the presented results, even parallel plating is not able to guarantee bone union when the elbow joint is loaded whilst close to full flexion, especially for the lateral column (ROI L).

The presented assessment of the bone union conditions was conducted based on the values of permissible stabilization stiffness established by Steiner et al. [29]. It should be noted that the upper limit of IFM calculated this way was equal to 0.5 mm. This value is lower than that obtained in other studies [38,39]. However, it must be emphasized that the calculated limit of IFM results directly from the force value taken into consideration in the research (150 N). In fact, this load can increase significantly during the healing process, for example, as a result of improper rehabilitation or uncontrolled events. Moreover, the presented IFMs in unfavorable cases significantly exceeded the permissible values, especially in the case of their tangential components (Figure 7), reaching the level of 1.05–1.60 mm. This makes the risk of non-union very high. This effect especially occurs in cases where the fracture plane is approximately parallel to the plane of the load action, which makes it easy for bone fragments to slide against each other. In this context, all fracture gaps extending along the sagittal plane should be treated as particularly unfavorable.

Based on the presented results, we recommend that in the period before bone union, full flexion of the elbow joint should not be allowed, unless this movement is performed passively. When using a perpendicular plate configuration (especially the posteromedial version), an even wider range motion in the elbow joint should be restricted. This result can be correlated with the commonly observed complication of the elbow joint having a limited range of motion after fracture healing, usually limited to the range of 99° [15] to 110° [7]. The fear of fracture destabilization and necessity of reoperation likely lead to a preventive limitation of motion in the early stages of the treatment. However, movement is essential for the success of the final treatment since the elbow is intolerant to immobilization [11,12,13]. The presented analysis results show that active flexion/extension can be safe even when lifting a 1 kg weight, provided that appropriate rules are followed. When a parallel plate configuration is used, elbow flexion/extension should be limited to the range of 0–90 degrees. However, even such limited movement can be beneficial during early rehabilitation since muscle strength returns faster and the range of motion returns earlier when weight training is applied. A perpendicular configuration allows for early rehabilitation with the use of external loads in a more limited range of motion, with a flexion/extension angle in the range of 0–30°. This knowledge can result in the modification of rehabilitation protocols, allowing for the earlier application of external loads, which can be positive in view of clinical treatment results. In addition, a wider range of motion greater than the presented limits is still possible, but should be performed without any external load.

This study has several limitations which need to be acknowledged. A constant value for the elbow force was used for various flexion angles, whilst the joint reaction varies during flexion/extension. Varus/valgus loadings were also neglected. Bone screws were modeled as fully bonded to both the plate and the bone tissue, disregarding the risk of screws loosening. The analyses were limited to the chosen method of screw placement. In clinical practice, the surgeon may use other lengths, numbers, and placements of screws, which can change the stiffness of the bone fragment fixation. The present analysis correspond to the early stage of fracture healing when no union between bone fragments is present and with the specific implants configurations that have been described in our manuscript. The research concept was focused on finding the weak points of particular plate configurations rather than proving the reliability of bone fusion in other cases.

## 5. Conclusions

The assessment of the mutual displacement of bone fragments made it possible to find the weak point of particular plate configurations. The main conclusions are as follows:

(1) No plating configuration is able to ensure DHF union when the full range of motion in the elbow (0–145°) is allowed while holding a weight of 1 kg in the hand.
(2) Elbow flexion in the range of 0–90°, lifting a weight of 1 kg, is allowed when using parallel plating, which is a positive finding in view of early rehabilitation.
(3) Better conditions for fracture healing are ensured when parallel plating is used. Worse conditions occur when perpendicular plating is used, especially the posteromedial version. In this case, the active elbow flexion should be limited to about 30°.

## Figures and Tables

**Table 1:** Direction of JRF in sagittal plane in the humeroulnar joint for the whole range of elbow flexion.

Angle of the Elbow Flexion	0°	* ^1^	30°	60–90°	120°	145°
JRF direction	−20°	0°	10°	43°	63°	95°

^1^ The symbol * indicates the unknown elbow position ensuring the direction of the JRF in agreement with the long humeral axis (JRF = 0°).

**Table 2:** Assessment of the biomechanical conditions of bone union in the case of distal humerus fracture based on the criteria presented in Figure 6. Non-union is highlighted in red, probable non-union is highlighted in orange, high probability of achieving bone union is highlighted in yellow, while optimal conditions for union are highlighted in green.

			Plating Configuration		
Region (ROI)	Joint Reaction	Joint Angle	PM	PL	Parallel
λ Fracture					
T	−20	0°	OPT	PU	PU
	10	30°	PU	PU	PU
	43	60–90°	PU	PU	PU
	63	120°	PU	PU	PU
	95	145°	PU	OPT	PU
S	−20	0°	PU	OPT	OPT
	10	30°	OPT	OPT	OPT
	43	60–90°	PNU	OPT	PU
	63	120°	NU	NU	PU
	95	145°	NU	NU	NU
M	−20	0°	PU	PU	PU
	10	30°	PU	PU	PU
	43	60–90°	PNU	PU	OPT
	63	120°	NU	PNU	OPT
	95	145°	NU	PNU	PNU
L	−20	0°	PU	PU	PU
	10	30°	PU	OPT	PU
	43	60–90°	PNU	OPT	PU
	63	120°	NU	NU	PNU
	95	145°	NU	NU	NU
H Fracture					
T	−20	0°	PU	OPT	OPT
	10	30°	PU	OPT	PU
	43	60–90°	PU	PU	PU
	63	120°	OPT	PU	PU
	95	145°	OPT	PU	PU
M	−20	0°	PU	OPT	PU
	10	30°	PU	OPT	PU
	43	60–90°	PNU	OPT	PU
	63	120°	NU	PNU	PNU
	95	145°	NU	NU	NU
L	−20	0°	PU	PU	PU
	10	30°	PU	PU	OPT
	43	60–90°	PNU	OPT	OPT
	63	120°	NU	NU	NU
	95	145°	NU	NU	NU
S	−20	0°	PU	PU	PU
	10	30°	PU	PU	PU
	43	60–90°	PNU	PU	PU
	63	120°	NU	NU	PNU
	95	145°	NU	NU	NU
Y Fracture					
T	−20	0°	PU	PU	OPT
	10	30°	PU	PU	OPT
	43	60–90°	PU	OPT	PU
	63	120°	PU	PU	PU
	95	145°	OPT	PU	PU
M	−20	0°	PU	PU	PU
	10	30°	PU	PU	PU
	43	60–90°	NU	PU	PU
	63	120°	NU	NU	PNU
	95	145°	NU	NU	NU
L	−20	0°	PU	PU	PU
	10	30°	OPT	PU	PU
	43	60–90°	OPT	PU	PU
	63	120°	NU	NU	NU
	95	145°	NU	NU	NU

## Data Availability

The datasets generated during and/or analyzed during the current study are available from the corresponding author on reasonable request.

## Supplementary Materials

The following supporting information can be downloaded at: https://www.mdpi.com/article/10.3390/jcm13041034/s1, Table S1: Mutual displacements of bone blocks in the fractured distal humerus.

## References

[1] Court-Brown C.M., Clement N.D., Duckworth A.D., Biant L.C., McQueen M.M. (2017). The changing epidemiology of fall-related fractures in adults. Injury.

[2] Bergdahl C., Ekholm C., Wennergren D., Nilsson F., Möller M. (2016). Epidemiology and patho-anatomical pattern of 2,011 humeral fractures: Data from the Swedish Fracture Register. BMC Musculoskelet. Disord..

[3] Claessen F.M.A.P., Braun Y., van Leeuwen W.F., Dyer G.S., van den Bekerom M.P.J., Ring D. (2016). What factors are associated with a surgical site infection after operative treatment of an elbow fracture?. Clin. Orthop. Relat. Res..

[4] Goel D.P., Pike J.M., Athwal G.S. (2010). Open reduction and internal fixation of distal humerus fractures. Oper. Tech. Orthop..

[5] Steinitz A., Sailer J., Rikli D. (2016). Distal humerus fractures: A review of current therapy concepts. Curr. Rev. Musculoskelet. Med..

[6] Rueadi T.P., Murphy W.E. (2009). AO Principle of Fracture Management.

[7] Yetter T.R., Weatherby P.J., Somerson J.S. (2021). Complications of articular distal humeral fracture fixation: A systematic review and meta-analysis. J. Shoulder Elb. Surg..

[8] Han S.H., Park J.S., Baek J.H., Kim S., Ku K.H. (2022). Complications associated with open reduction and internal fixation for adult distal humerus fractures: A multicenter retrospective study. J. Orthop. Surg. Res..

[9] Patel S.S., Mir H.R., Horowitz E., Smith C., Ahmed A.S., Downes K., Nydick J.A. (2022). ORIF of distal humerus fractures with modern pre-contoured implants is still associated with a high rate of complications. Indian J. Orthop..

[10] Vauclair F., Goetti P., Nguyen N.T., Sanchez-Sotelo J. (2020). Distal humerus nonunion: Evaluation and management. EFORT Open Rev..

[11] Leung B., McKee M., Peach C., Matthews T., Arnander M., Moverley R., Murphy R., Phadnis J. (2022). Elbow arthroplasty is safe for the management of simple open distal humeral fractures. J. Shoulder Elb. Surg..

[12] Ku K.H., Baek J.H., Kim M.S. (2022). Risk Factors for Non-Union after Open Reduction and Internal Fixation in Patients with Distal Humerus Fractures. J. Clin. Med..

[13] Savvidou O.D., Zampeli F., Koutsouradis P., Chloros G.D., Kaspiris A., Sourmelis S., Papagelopoulos P.J. (2018). Complications of open reduction and internal fixation of distal humerus fractures. EFORT Open Rev..

[14] O’Driscoll S.W. (2005). Optimizing stability in distal humeral fracture fixation. J. Shoulder Elb. Surg..

[15] Sanchez-Sotelo J., Torchia M.E., O’Driscoll S.W. (2008). Complex distal humeral fractures: Internal fixation with a principle-based parallel-plate technique. J. Bone Joint Surg. Am..

[16] Got C., Shuck J., Biercevicz A., Paller D., Mulcahey M., Zimmermann M., Blaine T., Green A. (2012). Biomechanical comparison of parallel versus 90-90 plating of bicolumn distal humerus fractures with intra-articular comminution. J. Hand Surg. Am..

[17] Jung S.W., Kang S.H., Jeong M., Lim H.S. (2016). Triangular fixation technique for bicolumn restoration in treatment of distal humerus intercondylar fracture. Clin. Orthop. Surg..

[18] Penzkofer R., Hungerer S., Wipf F., Oldenburg G., Augat P. (2010). Anatomical plate configuration affects mechanical performance in distal humerus fracture. Clin. Biomech..

[19] Schwartz A., Oka R., Odell T., Mahar A. (2006). Biomechanical comparison of two different periarticular plating systems for stabilization of complex distal humerus fracture. Clin. Biomech..

[20] Arnander M.W., Reeves A., MacLeod I.A., Pinto T.M., Khaleel A.A. (2008). Biomechanical comparison of plate configuration in distal humerus fractures. J. Orthop. Trauma.

[21] Zalavras C.G., Papasoulis E. (2018). Intra-articular fractures of the distal humerus-a review of the current practice. Int. Orthop..

[22] Koonce R.C., Baldini T.H., Morgan S.J. (2012). Are conventional reconstruction plates equivalent to precontoured locking plates for distal humerus fracture fixation? A biomechanics cadaver study. Clin. Biomech..

[23] Shin S.J., Sohn H.S., Do N.H. (2010). A clinical comparison of two different double plating methods for intraarticular distal humerus fracture. J. Shoulder Elb. Surg..

[24] Puchwein P., Wildburger R., Archan S., Guschla M., Tanzer K., Gumpert R. (2011). Outcome of type C (AO) distal humeral fractures: Follow-up of 22 patients with bicolumnar plating osteosynthesis. J. Shoulder Elb. Surg..

[25] Reising K., Hauschild O., Strohm P.C., Suedkamp N.P. (2009). Stabilization of articular fractures of the distal humerus: Early experience with a novel perpendicular plate system. Injury.

[26] Schmidt-Horlohe K.H., Bonk A., Wilde P., Becker L., Hoffmann R. (2013). Promising results after a treatment of simple and complex distal humerus type C fractures by angular-stable double-plate osteosynthesis. J. Orthop. Trauma.

[27] Varady P., Rüden C., Greinwald M., Hungerer S., Pätzold R., Augat P. (2017). Biomechanical comparison of anatomical plating systems for comminuted distal humeral fractures. Int. Orthop..

[28] Zha Y., Hua K., Huan Y., Chen C., Sun W., Ji S., Xiao D., Gong M., Jiang X. (2023). Biomechanical comparison of three internal fixation configurations for low transcondylar fractures of the distal humerus. Injury.

[29] Steiner M., Claes L., Ignatius A., Simon U., Wehner T. (2014). Numerical Simulation of Callus Healing for Optimization of Fracture Fixation Stiffness. PLoS ONE.

[30] Jupiter J.B., Mehne D.K. (1992). Fractures of the distal humerus. Orthopedics.

[31] Kincaid B.L., An K.N. (2013). Elbow joint biomechanics for preclinical evaluation of total elbow prosthese. J. Biomech..

[32] Sawbones. https://www.sawbones.com/catalog/biomechanical.html?cat=72_composite-bones.

[33] Claes L., Meyers N., Schülke J., Reitmaier S., Klose S., Ignatius A. (2018). The mode of interfragmentary movement affects bone formation and revascularization after callus distraction. PLoS ONE.

[34] Augat P., Hollensteiner M., von Rüdenb C. (2021). The role of mechanical stimulation in the enhancement of bone healing. Injury.

[35] Luciani A.M., Baylor J., Akoon A., Grandizio L.C. (2023). Controversies in the Management of Bicolumnar Fractures of the Distal Humerus. J. Hand Surg..

[36] Helfet D.L., Kloen P., Anand N., Rosen H.S. (2003). Open reduction and internal fixation of delayed unions and nonunions of fractures of the distal part of the humerus. J. Bone Joint Surg..

[37] Ring D., Jupiter J.B. (2002). Fracture–dislocation of the elbow. Hand Clin..

[38] Ferrara F., Biancardi E., Touloupakis G., Bibiano L., Ghirardelli S., Antonini G., Crippa C. (2019). Residual interfragmentary gap after intramedullary nailing of fragility fractures of the humeral diaphysis: Short and midterm term results. Acta Biomed..

[39] Baltov A., Mihail R., Dian E. (2014). Complications after interlocking intramedullary nailing of humeral shaft fractures. Injury.

